# Precision Medicine in Action: Multi-Tracer PET/CT Uncovers Tumor Heterogeneity and a Synchronous Malignancy

**DOI:** 10.1055/s-0045-1813676

**Published:** 2025-11-23

**Authors:** Siven Kar, Harshita Gupta, Vikram R. Lele, Nusrat Shaikh

**Affiliations:** 1 Department of Nuclear Medicine and PET/CT, Jaslok Hospital and Research Centre, Mumbai, Maharashtra, India

**Keywords:** multi-tracer PET/CT, tumor heterogeneity, precision oncology, neuroendocrine differentiation, nuclear medicine

## Abstract

Precision oncology leverages molecular profiling to tailor cancer treatment, with nuclear medicine playing an increasingly central role, particularly in elderly patients, where noninvasive approaches are preferable. We present a case of a 76-year-old male with metastatic prostate carcinoma (Gleason score: 5 + 5 = 10) exhibiting neuroendocrine differentiation. Imaging with multiple positron emission tomography (PET) tracers— ^68^ Ga-PSMA, ^18^ F-FDG, ^68^ Ga-DOTATATE, and ^68^ Ga-FAPI—revealed significant tumor heterogeneity. Tumor heterogeneity is multifactorial, influenced not only by genetic changes but also by epigenetic, microenvironmental, and therapeutic pressures. While ^68^ Ga-PSMA PET/computed tomography (CT) showed variable expression in osteosclerotic lesions, ^18^ F-FDG PET/CT detected metabolic activity, and ^68^ Ga-DOTATATE PET/CT confirmed low somatostatin receptor expression. Despite ^177^ Lu-PSMA radioligand therapy, the disease progressed with rising prostate-specific antigen levels. Subsequent gastrointestinal symptoms led to the diagnosis of sigmoid colon adenocarcinoma, confirmed by ^68^ Ga-FAPI PET/CT. This case exemplifies the critical role of nuclear imaging in characterizing tumor biology, guiding therapeutic strategies, and reducing reliance on invasive procedures. The integration of multi-tracer PET imaging enabled a nuanced understanding of tumor evolution, reinforcing nuclear medicine's importance in precision oncology and the management of complex, heterogeneous malignancies.

## Introduction

Precision oncology involves molecular and genomic profiling to identify specific tumor alterations for tailored therapies, impacting diagnosis, prognosis, and treatment efficacy. Tumor heterogeneity, driven by genetic, epigenetic, microenvironmental, and therapy-induced changes over time, poses challenges for accurate representation via single biopsies during staging. Nuclear medicine plays a pivotal role in this field, utilizing a range of radiotracers in positron emission tomography (PET) imaging for both diagnostic and therapeutic purposes. PET imaging provides insights into tumor heterogeneity, disease staging, treatment response assessment, and prognosis, potentially reducing the need for invasive procedures, particularly beneficial for elderly patients. This case is novel for demonstrating the simultaneous use of four PET tracers to reveal marked tumor heterogeneity in a neuroendocrine-differentiated prostate cancer with a synchronous colorectal malignancy.

## Case Presentation

A 76-year-old male, a known case of metastatic carcinoma of the prostate (Gleason score: 5 + 5 = 10, grade group 5 with neuroendocrine differentiation) for 1 year, initially received Abiraterone and monthly zoledronic acid injection. He presented to us with rising prostate-specific antigen (PSA) levels (137 ng/mL, reference range: <4 ng/mL).

At 12 months, ^68^ Gallium-prostate–specific membrane antigen PET/computed tomography ( ^68^ Ga-PSMA PET/CT) showed extensive osteosclerotic lesions, some of which showed moderate- to high-grade PSMA expression, while others were nontracer avid. There was low-grade diffuse PSMA expression in the prostate gland. He was advised for ^177^ Lutetium PSMA radioligand therapy ( ^177^ Lu-PSMA RLT).

An ^18^ F-fluorodeoxyglucose PET/CT ( ^18^ F-FDG PET/CT) was done. It showed mild- to moderate-grade metabolic activity in the multiple osteosclerotic lesions. There was no significant metabolic activity noted in the prostate. ^68^ Ga-DOTATATE PET/CT was done in view of neuroendocrine differentiation. It showed low-grade somatostatin receptor (SSTR) expression in the multiple osteosclerotic lesions.

He received three cycles of 200 mCi ^177^ Lu-PSMA RLT every 8 weeks. There was progression in PSA levels despite being on radioligand therapy and Apalutamide.

At 16 months, he then presented with complaints of lower abdominal pain, loss of appetite, and blood in stool. Colonoscopy revealed an ulcerative lesion 14 cm from the anal verge. Serum carcinoembryonic antigen levels were raised (102.6 ng/mL, normal 0–2.5 ng/mL). Biopsy revealed adenocarcinoma with focal signet ring cell morphology. He was advised for ^68^ Ga-fibroblast activation protein inhibitor PET/CT ( ^68^ Ga-FAPI PET/CT) in view of signet ring cell carcinoma. It showed high-grade fibroblast activation protein (FAP) expression in the heterogeneously enhancing circumferential wall thickening in the sigmoid colon with surrounding serosal irregularity and few tiny pericolonic lymph nodes. Low-grade FAP expression was seen in the known metastatic osteosclerotic lesions from primary prostate malignancy.

## Discussion

Precision oncology is evolving rapidly. It is the profiling of tumors at the molecular and genomic level to identify targetable alterations. 1 It can have diagnostic, predictive, prognostic, and therapeutic implications. Tumors are known to be heterogeneous, showing distinct phenotypic and functional profiles, arising from the cancer cells with the natural progression of the disease as it accumulates genetic changes. Single biopsies may not adequately capture this diversity, as they cannot represent all subclones within primary or metastatic lesions. 2

Nuclear medicine with its vast array of radiotracers plays an important part in this field, for imaging as well as therapeutic purposes. Each PET tracer provides unique information—on staging, restaging, dedifferentiation, and prognosis—helping to assess tumor heterogeneity without repeated biopsies, especially in older patients. 3 4

**Fig. 1:**
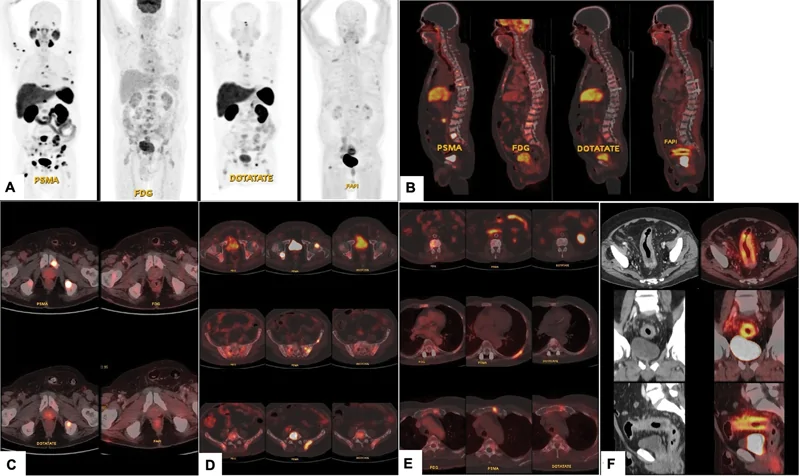
MIP images ( **A** ) showing multiple focal areas of tracer uptake with varying intensity on PSMA, FDG, DOTATATE, and FAPI. Sagittal ( **B** ) and axial ( **D, E** ) images showing high-grade PSMA expression in some osteosclerotic lesions, while others are nontracer avid; FDG showed moderate metabolic activity in multiple osteosclerotic lesions, including those that were non-PSMA avid; DOTATATE showing mild SSTR expression in the high-grade PSMA expressing lesions. Axial images of the prostate ( **C** ) showing no significant uptake by any tracer. ^68^ Ga-FAPI PET/CT axial, coronal, and sagittal images ( **F** show intense uptake in the concentric wall thickening of sigmoid colon and low-grade uptake in the known metastatic osteosclerotic lesions from primary prostate malignancy. CT, computed tomography; FDG, 18F-fluorodeoxyglucose; MIP, maximum intensity projection; PET, positron emission tomography; SSTR, somatostatin receptor.

**Fig. 2:**
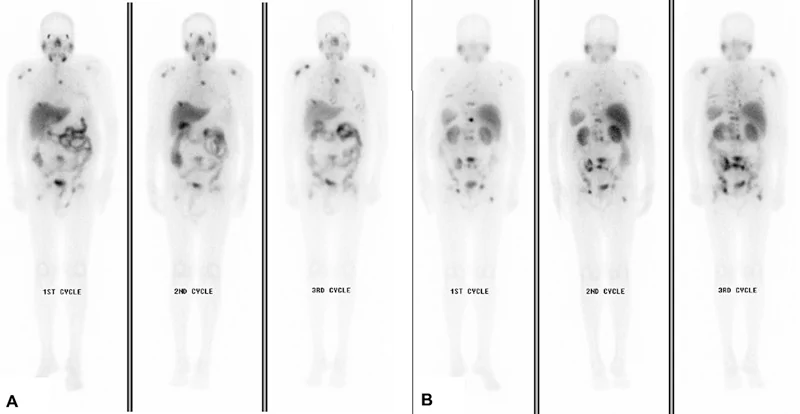
Post- ^177^ Lu-PSMA radioligand therapy scans: ( **A** ) anterior and ( **B** ) posterior images showing disease progression after two cycles of therapy with new-onset metastatic skeletal lesions.

Here, we present an interesting case of prostate adenocarcinoma with neuroendocrine differentiation (PCND) and signet cell carcinoma sigmoid colon, imaged with four different radiotracers of PET—PSMA, FDG, DOTATATE, and FAPI ( Fig. 1 )—and treated with three cycles of ^177^ Lu-PSMA RLT ( Fig. 2 ). PCND can present as untreated pathology or more commonly secondary to disease progression as resistance develops to androgen deprivation therapy. 5 6 PCND can further progress to neuroendocrine prostate carcinoma (NEPC). As NEPCs lose PSMA expression, FDG or SSTR imaging can be used as alternatives. 7 8

In our case, skeletal lesions showed heterogeneous uptake: some with high PSMA expression, others non-avid but FDG-positive. A few lesions also showed mild SSTR and FAP expression.

This case suggests that early use of novel tracers such as FAPI in patients with atypical or discordant progression may help avoid diagnostic delays and uncover synchronous malignancies, consistent with recent reports demonstrating the broad clinical applicability of FAPI across cancers and its superior lesion detection compared to FDG in certain contexts. 9 10 A limitation of this report is that it describes a single patient, and prospective studies are needed to validate these findings and define clear clinical workflows.

## Conclusion

This case highlights the pivotal role of nuclear medicine in the management of advanced prostate cancer with neuroendocrine differentiation, emphasizing its capability to assess tumor heterogeneity and guide therapy without the need for invasive biopsies, particularly in elderly patients. The use of multiple PET tracers, such as PSMA, FDG, DOTATATE, and FAPI, provided comprehensive insights into the diverse molecular profiles of metastatic lesions, aiding in treatment decision-making. This approach underscores the evolving landscape of precision oncology, where nuclear imaging not only informs diagnosis and staging but also influences therapeutic strategies tailored to individual tumor characteristics. As such, nuclear medicine continues to demonstrate its critical significance in enhancing patient care through personalized and targeted approaches in oncology. This case illustrates how multi-tracer PET can be integrated into clinical workflows to individualize therapy in heterogeneous malignancies.
